# Uncovering the connection between ceiling height and emotional reactions in art galleries with editable 360-degree VR panoramic scenes

**DOI:** 10.3389/fpsyg.2023.1284556

**Published:** 2023-12-21

**Authors:** Zhihui Zhang, Josep M. Fort, Lluis Giménez Mateu, Yuwei Chi

**Affiliations:** Escola Tècnica Superior d'Arquitectura de Barcelona, Universitat Politècnica de Catalunya, Barcelona, Spain

**Keywords:** ceiling height, editable 360-VR panoramas, emotional response, art galleries, environmental psychology, architectural design, virtual reality (VR)

## Abstract

**Introduction:**

This study investigates the relationship between ceiling height and emotional responses in art galleries, using editable 360-degree VR panoramic scenes. Prior research has explored the influence of spatial dimensions on general emotions, but the specific impact of ceiling height in art gallery settings, particularly on discrete emotions, remains understudied.

**Methods:**

The study utilized 360-degree panoramic photo scene modeling to modify ceiling heights within virtual art galleries, assessing emotional responses through self-report measures. Participants were presented with virtual art gallery environments featuring varying ceiling heights. Two studies were conducted: Study 1 involved absolute emotion rating across different ceiling heights, and Study 2 focused on selecting ceiling heights based on assigned emotions.

**Results:**

The data revealed that ceiling height significantly impacts specific emotions, notably disgust and joy. Lower ceiling heights generally evoked higher levels of fear and anger, while higher ceiling heights were associated with increased joy. The impact on other emotions like sadness, surprise, and disgust was more nuanced and varied across different ceiling heights.

**Discussion:**

The findings highlight a complex relationship between ceiling height and emotional responses in art galleries. The study demonstrates the efficacy of using editable 360-degree VR panoramic scenes in environmental psychology and architecture research, offering insights into how spatial dimensions influence emotional experiences in architectural settings.

## 1 Introduction

The built environment profoundly influences individual perceptions and experiences of spaces. Specific architectural characteristics, such as ceiling height and noise, influence occupant emotions and wellbeing (Reddy et al., 2012; Vartanian et al., 2013; Lyu and Kim, 2017; Soltani and Kirci, 2019; Mir et al., 2022). Acknowledging the role of architecture in evoking emotions has become a topic of increasing interest, contributing to the creation of spaces that stimulate positive experiences and enhance quality of life (Zeisel, 2006; Pallasmaa, 2011; Lee, 2022). This focus on emotional dimensions of architecture is part of a wider trend that aims to cultivate environments promoting human wellbeing (Altomonte et al., 2020).

Art galleries, museums, and other exhibition spaces are environments where emotional experiences are closely intertwined with architectural design, as the presentation of artworks or exhibits and the spatial dimensions of these spaces can affect visitors' emotional responses (Alelis et al., 2013; Kühnapfel et al., 2023). Understanding the relationship between ceiling height and emotional response in these settings can help architects create spaces that better cater to the needs of the exhibited items and enhance visitors' emotional experiences.

Virtual reality (VR) has emerged as a powerful tool for studying architectural experiences, offering researchers the capability to create immersive, controlled environments in which participants can explore and react to various design features (Kuliga et al., 2015). The 360-degree panoramic photo scene modeling technology, in particular, offers a level of control similar to 3D modeling techniques (Luo et al., 2019). In our study, we utilize this technology to specifically modify ceiling heights within the virtual art galleries, enabling us to examine their impact on occupants' emotional responses.

In this study, we aim to assess the relationship between ceiling height and emotional response in art galleries using editable 360-degree VR panoramic scenes. By examining how different ceiling heights affect participants' emotions, we hope to contribute to the broader understanding of the role of spatial dimensions in architectural design and their influence on emotional experiences. Additionally, the use of innovative VR technology will allow us to explore the potential of editable 360-degree panoramic photo scene modeling as a valuable research tool in the field of architecture and design.

This paper is organized as follows: First, we review relevant literature on the relationship between architectural features and emotional responses, with a particular focus on ceiling height. Second, we describe the methodology used in our study, including the creation of editable 360-degree VR panoramic scenes and the assessment of participants' emotional responses. Third, we present our findings, highlighting the specific emotions that were significantly impacted by ceiling height and discussing the potential influence of exhibited works on emotional responses within an art gallery context. Lastly, we conclude by summarizing our findings and proposing directions for future research.

By investigating the relationship between ceiling height and emotional response in art galleries through editable 360-degree VR panoramic scenes, we aim to provide valuable insights for architects, designers, and researchers interested in understanding the complex interplay between built environments and human emotions.

## 2 Literature review

The impact of architectural features on human emotions and cognition is an area of growing interest. This literature review focuses on the relationship between ceiling height and emotional responses, as well as the use of 360-degree panoramic photo scene modeling in architectural research. By examining existing studies and addressing research gaps, we aim to provide a foundation for our investigation into the influence of ceiling height on emotional response in art galleries through editable 360-degree VR panoramic scenes.

### 2.1 Perception and response to ceiling height

Ceiling height has been a subject of interest in architecture and environmental psychology, with numerous studies exploring its influence on human perception and behavior (Meyers-Levy and Rui, 2007; Guimarães et al., 2013; Mofrad, 2014; Vartanian et al., 2015; Cha et al., 2019). Recent research has begun to examine the connection between ceiling height and emotional response. This literature review delves into the perception of and response to ceiling height, providing a foundation for studying its impact on emotional responses in art galleries, specifically through 360-degree VR panoramic scenes.

The Cathedral Effect describes the relationship between perceived ceiling height and cognition, wherein high ceilings promote abstract thinking and creativity, while low ceilings encourage concrete and detail-oriented thinking (Meyers-Levy and Rui, 2007). Expanding on this concept, research has found that rooms with higher ceilings and open spaces are more likely to be judged as beautiful, activating brain structures related to visuospatial exploration, attention, and perceived visual motion, while enclosed rooms elicit exit decisions and activate emotional response regions in the brain (Vartanian et al., 2015). Building on this research, studies have found that participants in high-ceiling environments experience more positive emotions, while those in low-ceiling spaces report more negative emotions (Cha et al., 2019). These emotional differences are thought to be linked to the sense of spaciousness and freedom associated with high ceilings and the feelings of confinement related to low ceilings.

In terms of thermal comfort, studies have examined the influence of floor-to-ceiling height on human comfort in residential units, concluding that the ideal height falls between 2,700 mm and 2,800 mm (Mofrad, 2014). Furthermore, it has been demonstrated that a 20 cm decrease in ceiling height led to a 1°C temperature increase (Guimarães et al., 2013). Research utilizing a height-matching method to assess the perceived height of ceilings with varying lightness found that lighter ceilings appeared significantly higher than darker ones (von Castell et al., 2014). This method avoids metric judgments, indicating that the impact of ceiling lightness on perceived height is a direct perceptual effect rather than a cognitive one. Collectively, these studies emphasize the relevance of ceiling height on human perception and emotional response.

The literature on ceiling height and emotional response is expanding, with an increasing number of studies leveraging virtual reality (VR) technology. One of the primary reasons for the adoption of VR in these studies is the difficulty of manipulating ceiling height within the same physical space while controlling for other factors. In real-world environments, changing ceiling heights while maintaining other architectural elements constant is often challenging, if not impossible. Therefore, VR has emerged as one of the preferred tools for studying the relationship between ceiling height and emotional responses, as it allows researchers to create controlled environments where ceiling height can be easily manipulated while keeping other factors constant.

### 2.2 360-degree panoramic photo scene modeling

Virtual reality (VR) technology offers a solution to this issue by providing immersive and realistic environments, which in turn allows for a broader range of possibilities when studying the impact of architectural features on emotions. In emotion research using virtual reality, three main methods are typically employed to create virtual environments: computer-generated 3D models, 360-degree panoramic photos, and 360-degree panoramic videos.

Each of these methods has been proven effective in influencing emotions through various studies. For instance, one study developed an emotion recognition system for affective states evoked through immersive virtual environments using computer-generated 3D models, achieving accuracies of 75.00% for arousal and 71.21% for valence (Baños et al., 2006). Another study explored the effects of virtual nature exposure on subjective vitality using 360-degree panoramic photos, finding that individuals with high levels of cognitive reappraisal experienced significant and positive effects (Theodorou et al., 2023). Lastly, a comparison of emotions and sense of presence elicited by an immersive 360-degree video of a landscape to those evoked in a real-life contemplative scenario of the same landscape found that emotions and sense of presence in the virtual condition were largely comparable to those experienced in the real-life condition, with only minor differences in anger and amusement levels (Alelis et al., 2013).

In architectural perception and response research within virtual reality, researchers often use 3D models for controllable study environments or opt for 360-degree panoramic photos for increased realism at the expense of control. Recently, the emergence of 360-degree panoramic photo scene modeling has provided a balanced approach between realism and control for researchers studying ceiling height. This technique allows the manipulation and editing of 360-degree panoramic images, enabling the creation of virtual environments and augmented reality experiences. It combines panoramic photography and 3D modeling, creating an interactive and immersive experience. Panoramic texture mapping and panoramic 3D reconstruction are two essential technologies in the fields of computer graphics and computer vision, facilitating the creation of virtual environments and augmented reality experiences (Pintore et al., 2019; Walmsley and Kersten, 2020).

Panoramic texture mapping is a technique that maps 2D textures, such as panoramic images, onto a scene's 3D model, creating the illusion of a panoramic environment. This method has been widely used in virtual reality and video game applications, creating realistic and immersive environments (Zou et al., 2018; Luo et al., 2019; Pintore et al., 2021). On the other hand, panoramic 3D reconstruction involves using computer vision and machine learning algorithms to infer a scene's 3D structure from single or multiple panoramic images and create a 3D model of the scene (Liu et al., 2015; Nebiker et al., 2016; Xu et al., 2017). Panoramic texture mapping is simple and fast, requiring only basic image processing techniques. However, its performance is limited to the appearance of the environment and does not provide information about its geometry or depth (Pan et al., 2011). In contrast, panoramic 3D reconstruction captures both the appearance and geometric shape of the environment, creating a more comprehensive representation. This method requires more advanced techniques, such as machine learning algorithms, and is more time-consuming and computationally intensive. However, the current state of technology for panoramic 3D reconstruction has limitations in accurately reproducing the realism of the environment.

In summary, 360-degree panoramic photo scene modeling is a promising technique for researching architectural perception and response in virtual reality. This method combines the advantages of panoramic photography and 3D modeling, providing a balance between realism and control. This approach offers a technological foundation for creating editable 3D models using 360-degree panoramic photos.

### 2.3 Addressing the research gaps and formulating hypotheses

In the existing literature on the impact of ceiling height on human emotions, several research gaps have been identified, emphasizing the necessity for an enhanced approach. First, some studies rely on 3D models to simulate ceiling heights (Cha et al., 2019), which may not accurately represent real-world environments. Using 3D models might limit the ecological validity of research findings since they lack the authenticity and complexity of real-world spaces. Methods that better replicate real environments, such as editable 360-degree panoramic scenes, should be considered for simulating architectural settings.

Second, previous research has often focused on comparing only two ceiling heights or different heights with varying scenes (Vartanian et al., 2015; Cha et al., 2019), and the selection of these heights may lack a clear rationale. The limited range of ceiling heights studied might not fully capture the entire scope of potential impacts on human emotions. A more systematic investigation of different ceiling heights is needed, with clear rationales for their selection, to better understand the emotional impact of ceiling heights.

Moreover, most studies use absolute emotional values; however, emotions are influenced by multiple factors. As highlighted in a particular study (Liang et al., 2018), it is important to integrate both direct person-independent (absolute emotions) and relative person-dependent (relative emotions) approaches for emotion recognition, providing a more comprehensive understanding of the impact of ceiling heights on emotions. Based on these identified gaps, we propose the following hypotheses:

1. There is a significant relationship between ceiling height and emotional responses in art galleries, with higher ceiling heights eliciting more positive emotions and lower ceiling heights eliciting more negative emotions among gallery visitors.
2. Utilizing editable 360-degree panoramic scenes to simulate various ceiling heights in art galleries effectively replicates real-world environments, thereby inducing emotional changes in viewers. This approach will be used to investigate the impact of ceiling height variations on participants' emotional responses, demonstrating the efficacy of this technology as a tool for studying the influence of architectural dimensions on emotions.

## 3 Methodology

### 3.1 Scene selection rationale

In this study, we chose to use panoramic texture mapping technology for operability and realism. Due to the technical limitations of panoramic texture mapping, there are certain requirements for scene selection. First, rooms with isometric shapes, such as rectangular, circular, or hexagonal rooms, are preferred, as these rooms allow for a more even division of wall texture in panoramic photos. Second, spaces without furniture and decorations are selected, as the software's texture projection will cast any furniture or decorations onto the walls, affecting the final result's authenticity. Third, well-lit scenes with sufficient illumination are essential for taking panoramic photos.

**Figure 1:**
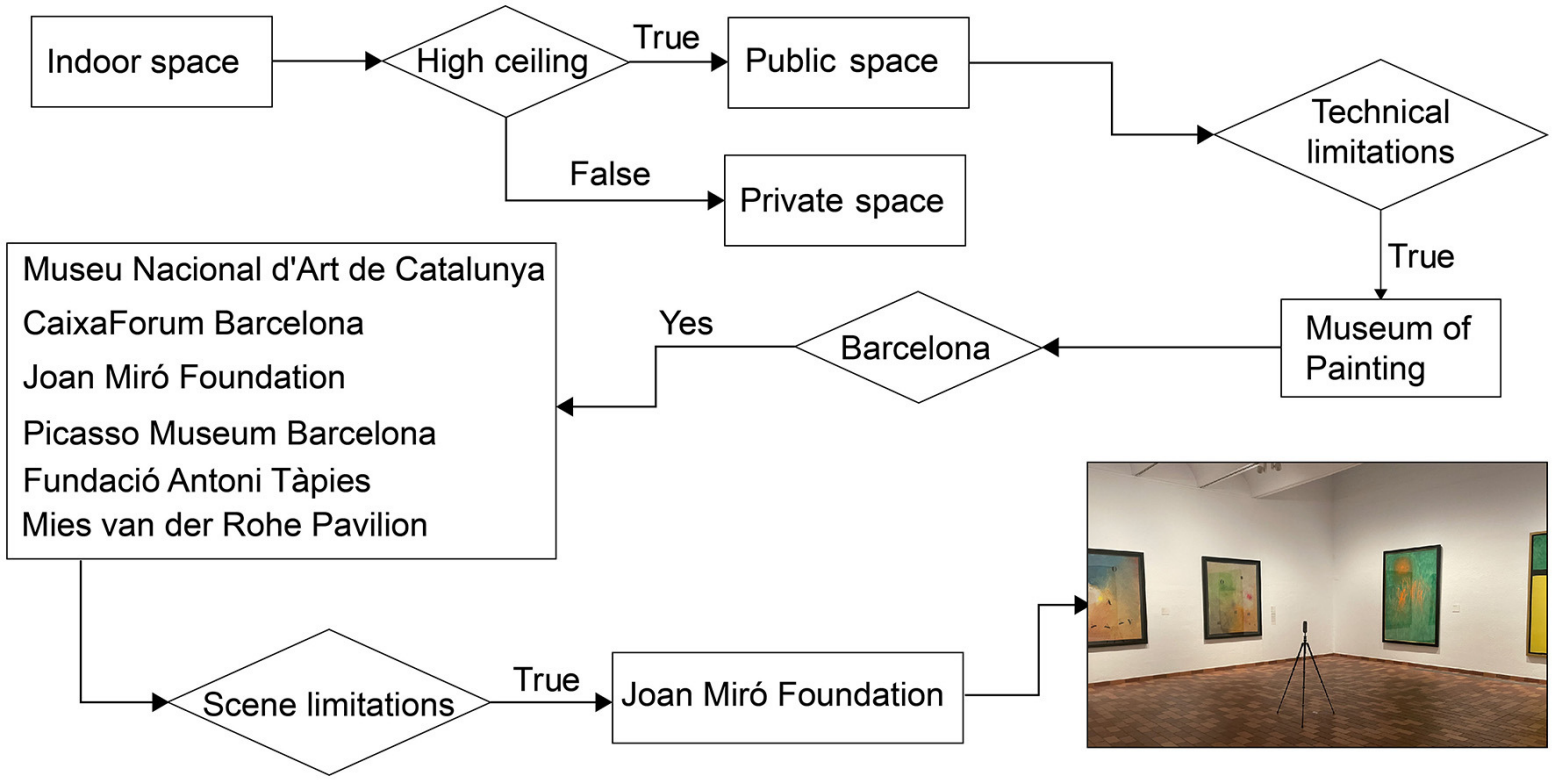
Scene selection rationale.

Selecting suitable scenes for our research is a crucial aspect of our study, and the following outlines our choice logic (see Figure 1). Given that residential spaces generally have little variation in height (Appolloni and D'alessandro, 2021), we chose to focus on public spaces. Taking into account the technical limitations previously mentioned, museums and galleries were deemed appropriate public spaces for our investigation. We visited various museums in Barcelona, including the Catalan National Art Museum, CaixaForum Barcelona, Joan Miró Foundation Museum, Picasso Museum, Fundació Antoni Tápies Museum, and Mies van der Rohe Pavilion.

In addition to considering technical requirements, we identified several factors to take into account during the scene editing process: (1) enclosed spaces, as the field of view changes when the room's dimensions change in open spaces; although artificial intelligence technology can fill in missing scenes, authenticity is still affected; (2) clear boundaries between walls and ceilings to facilitate software processing when adjusting ceiling heights; (3) single-material wall textures, as multi-material walls pose significant challenges for expanding the simulated texture mapping; (4) the absence of hanging objects, as changing ceiling heights would cause the perspective ratio of hanging objects to differ from reality, affecting authenticity. After filtering through these criteria, we chose a room within the Joan Miró Foundation Museum measuring approximately 8.11 meters by 9.21 meters, resembling a square shape, as our research space and obtained permission from the museum to conduct scene photographs.

### 3.2 Adopting Le Corbusier's Modulor system for ceiling height

**Figure 2:**
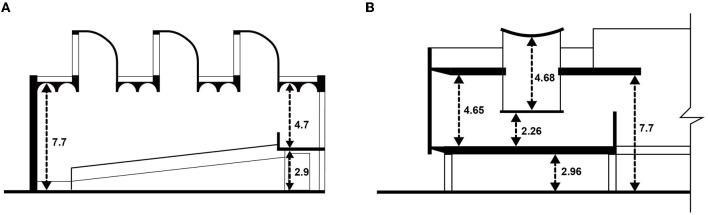
**(A)** Cross-sectional heights of Joan Miró Foundation, **(B)** cross-sectional heights of National Museum of Western Art.

To choose the ceiling heights for our study, we incorporated Le Corbusier's Modulor system, an innovative and human-centered approach to architectural design (Corbusier et al., 1980). This choice was guided by historical and empirical reasons. Notably, Josep Lluís Sert, the designer of the Joan Miró Foundation Museum, who had worked in Le Corbusier's studio, applied his design principles in his works (Bair, 2015). Upon measuring the ceiling heights of the Joan Miró Foundation Museum, we found that they corresponded to Le Corbusier's Modulor system. Similarly, the Tokyo National Museum of Western Art, a collaborative design by Le Corbusier and Kunio Maekawa, also adhered to the Modulor system (Yamana and Fukuda, 2015) (see Figure 2).

The Modulor system is a well-grounded, human-proportion-based approach to architectural design. This system not only ensures the heights chosen align with ergonomic considerations and spatial perception, but it also conforms to broader architectural discourse and practice. Consequently, the findings from our research are more directly applicable to real-world scenarios.

Given the human-centric focus of the Modulor system, we adopted the red and blue series for determining our experimental ceiling heights (Steyn, 2012; Itham Mahajan, 2016; Rozhkovskaya, 2020). The heights selected, namely, 2.26, 2.96, 3.66, 4.79, 5.92, 7.74, and 9.57 m, are found in both the Joan Miró Foundation Museum and the Tokyo National Museum of Western Art. For reference purposes, 4.79 m was chosen as it is approximately double the lowest height of 2.26 m and half of the highest height of 9.57 m. It's important to note that our experiment did not include ceiling heights lower than 2.26 m or higher than 9.57 m. In the context of public spaces such as art galleries and museums, we categorize the ceiling heights of 2.26 and 2.96 m as “lower,” while 3.66, 4.79, and 5.92 m are considered “common” heights. Conversely, 7.74 and 9.57 m represent “higher” ceiling heights. This categorization is grounded in our examination of ceiling heights in existing museum and gallery spaces, allowing us to present our findings within a relevant architectural context.

These heights, though might seem unconventional in some contemporary architectural projects, hold significant empirical value in that they follow the anthropocentric design principles of the Modulor system.

### 3.3 Designing environments with diverse ceiling heights

In the production process of our experimental scenes, we started by selecting an enclosed, rectangular exhibition room without exterior windows at the Joan Miró Foundation museum. To capture the 360-degree panoramic images, we positioned a Trisio Lite2 8K camera at the center of the room, ensuring a comprehensive view of the entire space. After acquiring the images, we utilized the Blender PanoCamAdder(+) plugin to intelligently project the captured scene onto the walls, and the arched ceiling, which allowed us to generate texture maps for each surface using the “unwarp” command.

**Figure 3:**
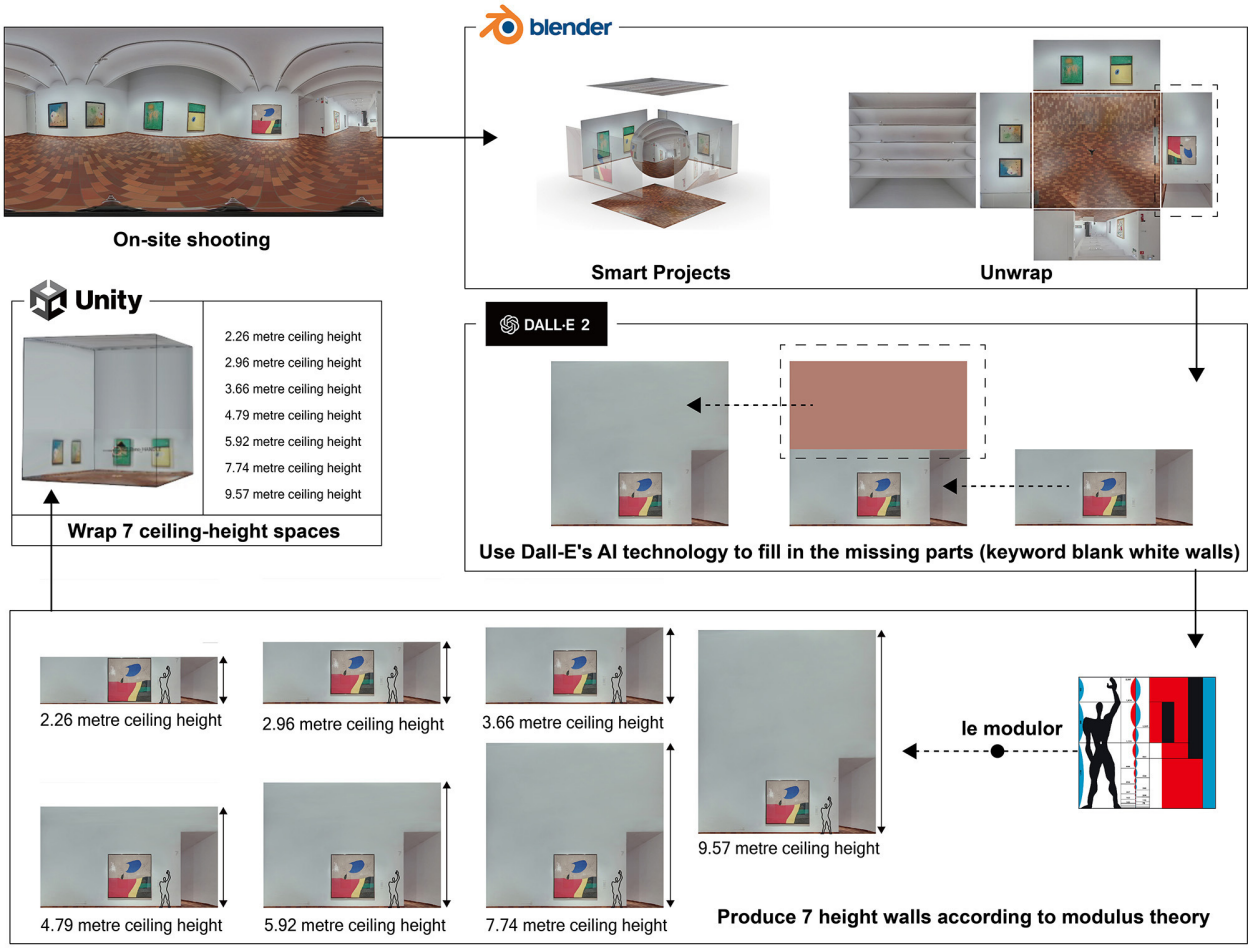
Process for designing environments with diverse ceiling heights.

To create the different ceiling heights according to Le Corbusier's Modulor theory, we employed OpenAI's Dall-E2 web application to generate the wall images required for each height (Reviriego and Merino-Gómez, 2022). We input the keyword “blank white walls” and selected the most suitable images from the generated results. We iterated the generation process if an image contained imperfections until the desired outcome was achieved. With the AI-generated images, we used Unity to reassemble the walls and create virtual room models for the seven ceiling heights (2.26, 2.96, 3.66, 4.79, 5.92, 7.74, and 9.57m) (see Figure 3). In preparation for the main study, we conducted pre-tests with a preliminary group of participants to validate the perceptual effectiveness of the virtual environments, particularly in terms of ceiling height variations. Their feedback was instrumental in fine-tuning the virtual spaces to ensure realistic and immersive experiences for the main study participants. This meticulous process allowed us to generate high-quality and accurate models for our experiment, ensuring a realistic and immersive experience for the participants while maintaining consistency across the different ceiling heights.

### 3.4 Questionnaire development

To ensure that participants were fully immersed in the VR environment and to maintain contextual event continuity, we developed a questionnaire that was embedded within the Unity-based VR experience (Parsons, 2015). This approach allowed participants to complete the questionnaire while remaining in the VR environment, preserving the ecological validity of their emotional responses to the architectural spaces.

**Figure 4:**
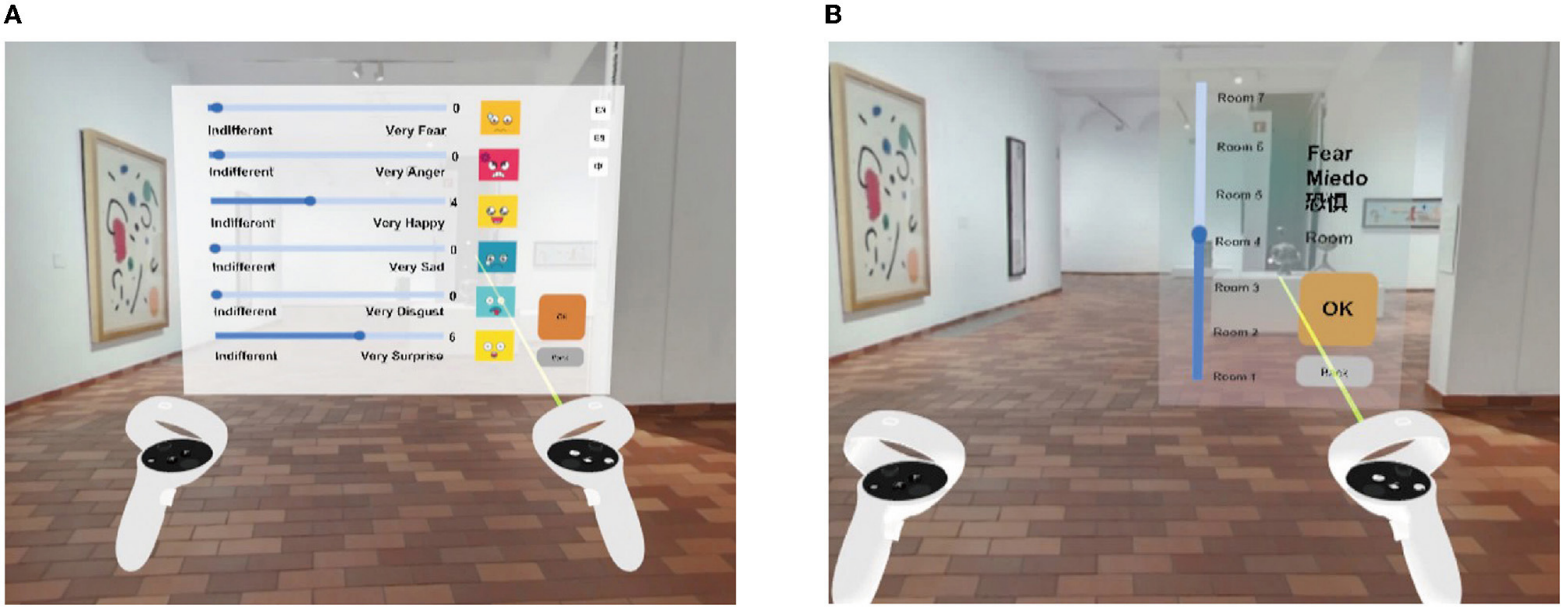
**(A)** VR-embedded questionnaire interface in Study 1, **(B)** the seven-room options with varying ceiling heights for participants to choose from in Study 2.

In Study 1, the VR-embedded questionnaire consisted of rating scales for each of Ekman's six basic emotions: fear, anger, joy, sadness, disgust, and surprise (Ekman, 1992) (see Figure 4A). Based on our previous research experience, we found that participants often struggled to understand complex emotional scales like the Self-Assessment Manikin (SAM) (Zhang et al., 2022). Additionally, in a VR setting, text-heavy questionnaires such as the Emotion Beliefs Questionnaire (EBQ) (Becerra et al., 2020) are not ideal due to the immersive nature of the environment. Therefore, we opted for a simpler, more direct emotional rating scale complemented by visual aids. Participants were asked to rate their emotional response to the assigned ceiling height on a scale from 0 to 10, with 0 representing “indifferent or neutral” and 10 representing a strong emotional response (e.g., “very fearful” for fear). This rating scale was presented to the participants within the VR environment after they had explored the architectural space for three minutes, ensuring that they had sufficient time to experience and react to the space.

In Study 2, participants were assigned an emotion from Ekman's six basic emotions and were asked to select a ceiling height that best resonated with their assigned emotion. They were presented with seven room options, each with a different ceiling height (2.26, 2.96, 3.66, 4.79, 5.92, 7.74, or 9.57m) (see Figure 4B). This task aimed to explore participants' perceptions of the relationship between ceiling heights and emotions.

### 3.5 Participants

A total of 183 participants from Universitat Politécnica de Catalunya, took part in both Study 1 and Study 2. All participants were architecture students, with a diverse mix of academic levels (i.e., undergraduate, graduate, and doctoral students). The age range of the participants was between 18 and 45 years old. Participants were divided into six groups based on the ceiling height they were assigned to in Study 1: 2.26 m (31 participants), 2.96 m (30 participants), 3.66 m (29 participants), 5.92 m (30 participants), 7.74 m (31 participants), and 9.57 m (32 participants). In Study 2, due to participant errors, only 178 responses were recorded. The groups for Fear, Joy, Sad, Disgust, and Surprise each consisted of 30 participants, while the Anger group had 28 participants.

No demographic information, such as gender, was recorded for this experiment in accordance with the principles outlined in the article “Stop ‘controlling' for sex and gender in global health research,” ensuring the focus remained on architectural perception (Shapiro et al., 2021). As the experimental spaces were designed with uniformly white walls, we did not exclude participants with color blindness. Participants with myopia were allowed to wear their corrective glasses during the study. Informed consent was obtained from all participants, and the study was conducted in compliance with ethical guidelines and principles.

### 3.6 Experimental procedures for Study 1 and Study 2

Before the experiment began, the researcher took 5 minutes to explain the procedure and the purpose of the study. Those who agreed to participate in the experiment were asked to sign the informed consent form. The total duration of the experiment was approximately 15 min.

The experiment consisted of two distinct studies, with each participant experiencing both. Initially, participants wore disposable VR eyes masks and VR headsets (Oculus Quest 2) to enter the virtual environment for Study 1. All participants began in a room with a ceiling height of 4.79 m, which served as the reference environment for within-subject comparisons. After exploring the room, they completed the first emotional assessment questionnaire within the VR space. Once the first emotional assessment was completed, participants were divided into six groups, each assigned to a different ceiling height (2.26, 2.96, 3.66, 5.92, 7.74, or 9.57 m), employing a between-subjects design. They were instructed to explore their assigned space and then complete the second emotional assessment using the same questionnaire as before. This approach allowed us to compare the emotional responses across different ceiling heights as well as to examine within-subject changes due to the alteration of ceiling heights.

**Figure 5:**
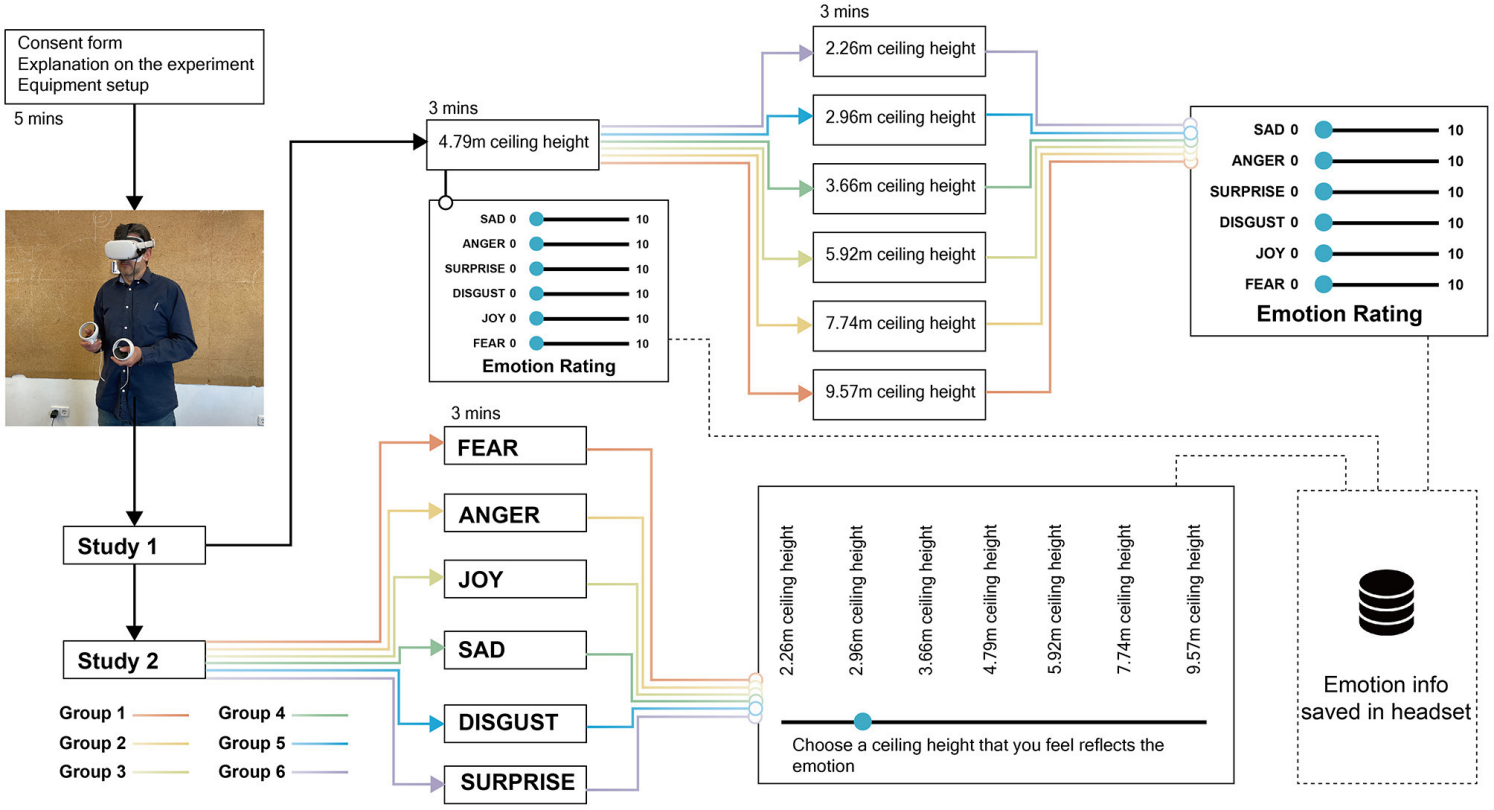
Flowchart of experimental procedures for Study 1 and Study 2.

For Study 2, participants were divided into six new groups corresponding to each of Ekman's six basic emotions. In this study, participants were tasked with selecting a ceiling height (2.26, 2.96, 3.66, 4.79, 5.92, 7.74, or 9.57 m) that they felt best resonated with their assigned emotion. This exercise aimed to explore the participants' perception of the relationship between ceiling heights and emotions (see Figure 5).

The experiment's data was stored on the VR headsets. Upon completion, researchers checked the equipment to ensure participants had followed the instructions correctly. The headsets were then disinfected according to safety protocols, ensuring a clean and safe environment for subsequent participants.

### 3.7 Statistical analysis

We first preprocessed the collected data, addressing two outliers resulting from participant errors during the experiment, following best practices for data cleaning and preprocessing. Next, we conducted a descriptive statistical analysis using Python to calculate the main features of the data, such as mean, standard deviation, and median, allowing us to better understand the data's distribution and trends.

In our data analysis, we employed different statistical methods depending on the distribution of the data. When the data adhered to a normal distribution, we used the *t*-test and computed Cohen's d to estimate the effect size (Schmidt and Bohannon, 1988). Conversely, for data that did not conform to a normal distribution, we implemented the Wilcoxon signed-rank test and Cliff's delta (Cliff, 1993). These methods were chosen to ensure a comprehensive and robust statistical analysis, regardless of the data's distribution (Bloice and Holzinger, 2016).

To facilitate our data processing and analysis, we utilized several Python packages. Specifically, we employed NumPy for numerical operations, Pandas for data manipulation and analysis, SciPy for implementing statistical tests, and Matplotlib along with Seaborn for data visualization. The use of these packages ensured rigorous and efficient processing of our experimental data, contributing to the reliability and reproducibility of our results.

## 4 Result

### 4.1 Study 1: absolute emotion rating

**Figure 6:**
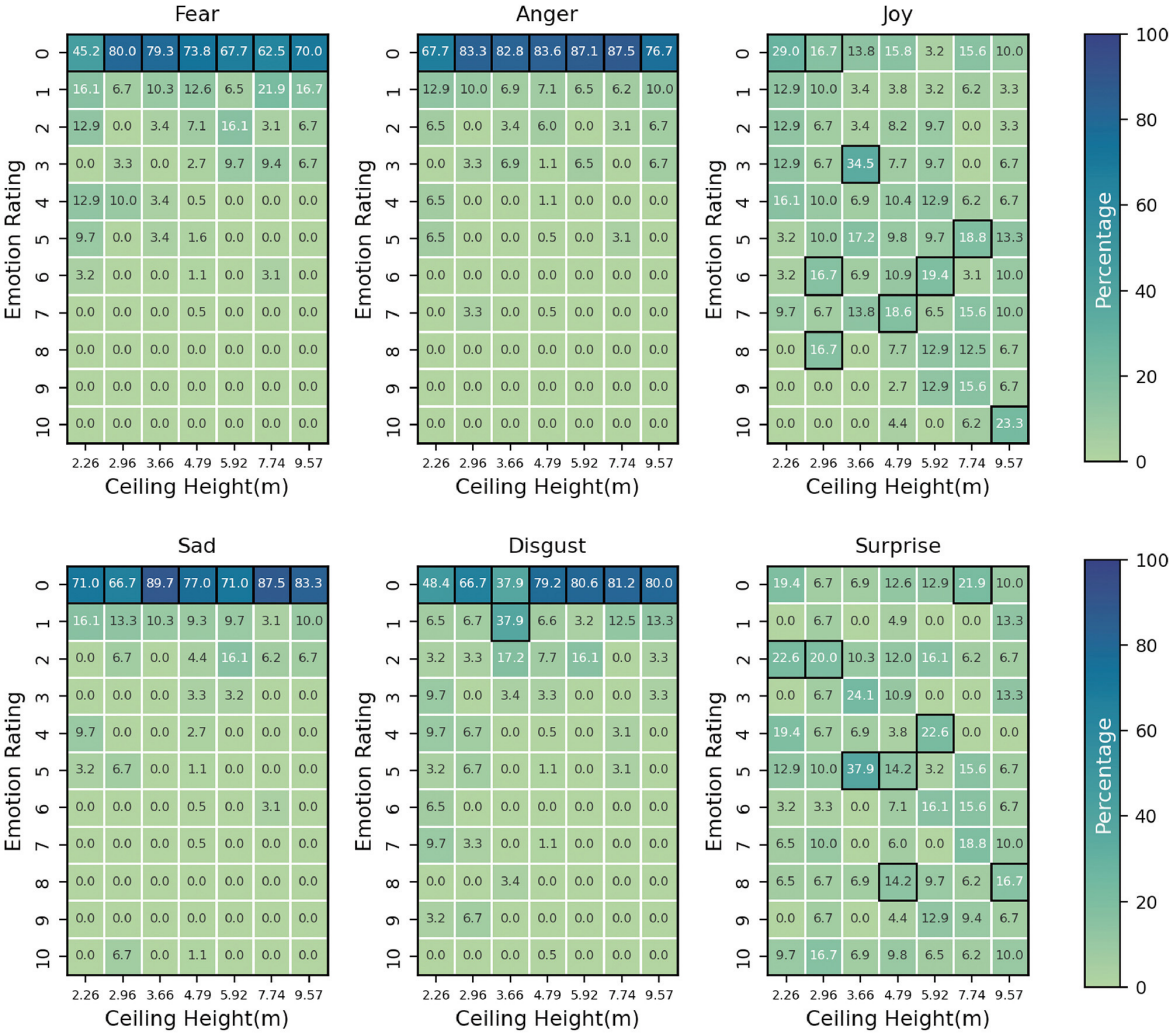
Heatmap of absolute emotion values for six basic emotions across ceiling heights.

This section presents a comprehensive analysis of the absolute emotional ratings across various ceiling heights, ranging from 2.26 to 9.57 m. The assessed emotions include fear, anger, joy, sadness, disgust, and surprise, with participants rating their emotions on a scale from 0 to 10. The key findings from the data are as follows (see Figure 6):

1. Negative emotions:
• Fear: Participants generally expressed higher levels of fear in rooms with lower ceiling heights. For instance, in the 2.26 m ceiling height, 45.16% of participants gave a rating of 0 for fear, while this proportion increased to 80% in the 2.96 m ceiling height. As the ceiling height increased, the proportion of participants who rated their fear as 0 decreased, reaching a minimum of 62.5% in the 7.74 m ceiling height. However, it rose slightly to 70% in the 9.57 m ceiling height.
• Anger: A similar trend can be observed for anger, where higher proportions of low ratings were observed in rooms with lower ceiling heights. In the 2.26 m ceiling height, 67.74% of participants gave a rating of 0, while this proportion increased to 83.33% in the 2.96 m ceiling height. The proportion of 0 ratings remained relatively high across all the other ceiling heights, with the lowest being 76.67% in the 9.57 m ceiling height.
• Sadness: Participants reported low levels of sadness across all ceiling heights. The proportion of 0 ratings was consistently high, ranging from 70.97% in the 2.26 m ceiling height to 89.66% in the 3.66 m ceiling height. The highest ceiling height, 9.57 m, still had a high proportion of 0 ratings (83.33%).
• Disgust: Disgust ratings were predominantly low across all ceiling heights. In the 2.26 m ceiling height, 48.39% of participants gave a rating of 0, while this proportion increased to 80.65% in the 5.92 m ceiling height. The 9.57m ceiling height had the highest proportion of 0 ratings (80%).
2. Positive emotions:
• Joy: The distribution of joy ratings across different ceiling heights was more varied. The proportion of 0 ratings was highest in the 2.26 m ceiling height (29.03%) and lowest in the 5.92 m ceiling height (3.23%). Interestingly, the 10 rating had the highest proportion in the 9.57 m ceiling height (23.33%), suggesting that participants experienced more joy in ceiling height with higher ceiling heights.
• Surprise: Surprise ratings exhibited a diverse distribution across various ceiling heights. The proportion of 0 ratings was highest in the 2.96 m ceiling height(6.67%) and lowest in the 5.92m ceiling height (12.90%). The 9.57 m ceiling height had a relatively high proportion of 0 ratings (10%) but also had a high proportion of 10 ratings (10%).

### 4.2 Study 1: relative emotion rating

The importance of relative emotion analysis is evident in our data. For instance, in the emotion “joy,” we can observe variations in reported intensity across different groups experiencing the same ceiling height. These variations, indicated by a range of mean values from 3.709 to 5.531, and standard deviations varying from 2.344 to 3.089, highlight the need for an analysis method that better accommodates for this intra-group variation, such as relative emotion analysis. To gain a more objective understanding of the participants' emotional responses, we employed a method that utilized relative emotional values. In this approach, we designated the 4.79-meter ceiling height as the reference height. We then compared the emotional responses from six comparison heights (2.26, 2.96, 3.66, 5.92, 7.74, and 9.57 m) to the responses at the reference height. This analysis aimed to identify differences and correlations between emotional responses in rooms with varying ceiling heights and the reference height.

**Figure 7:**
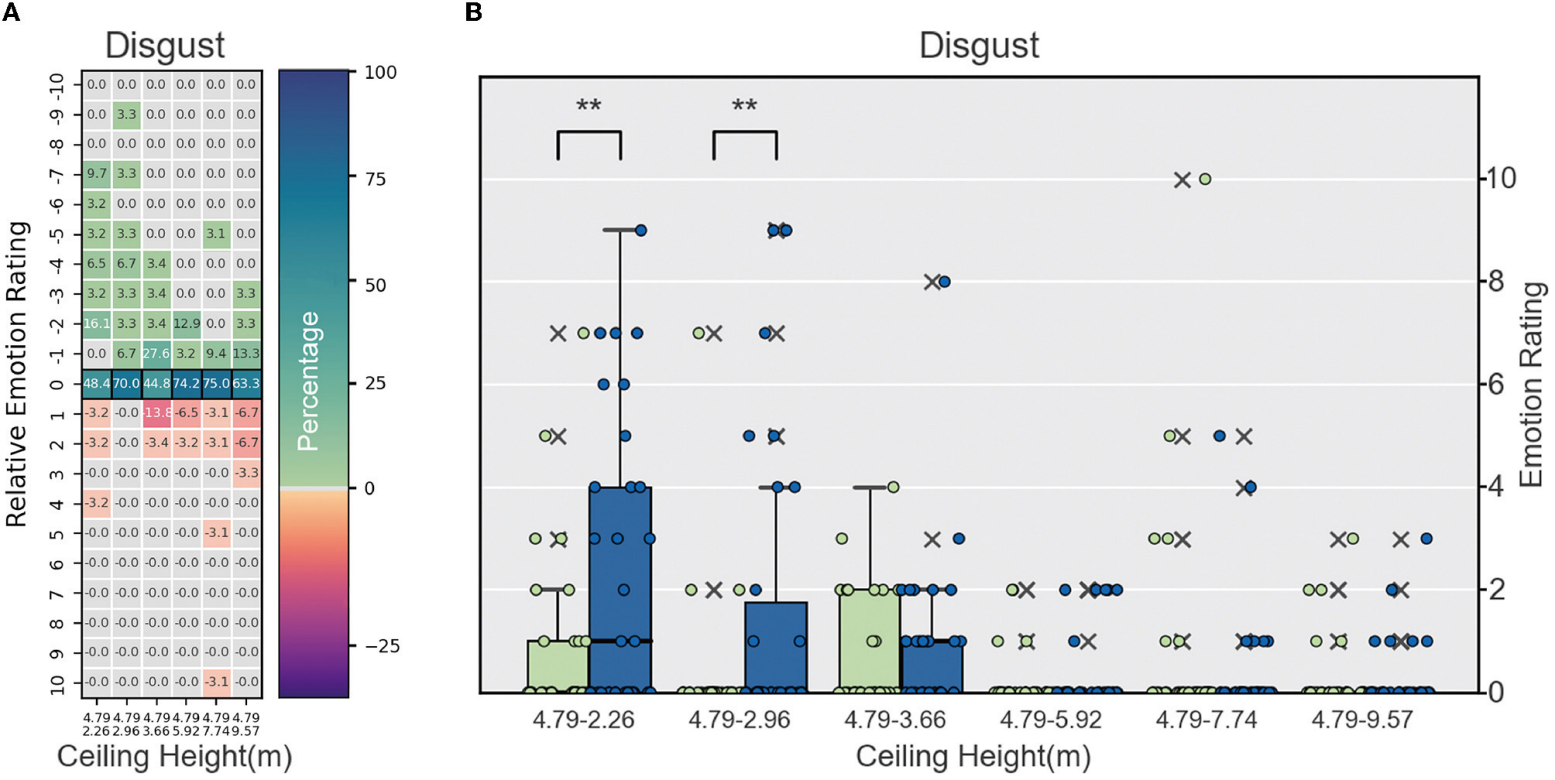
**(A)** Heatmap of relative emotion analysis for disgust across ceiling heights, **(B)** box plot comparing disgust responses at different ceiling heights to reference height, **p < 0.01.

**Figure 8:**
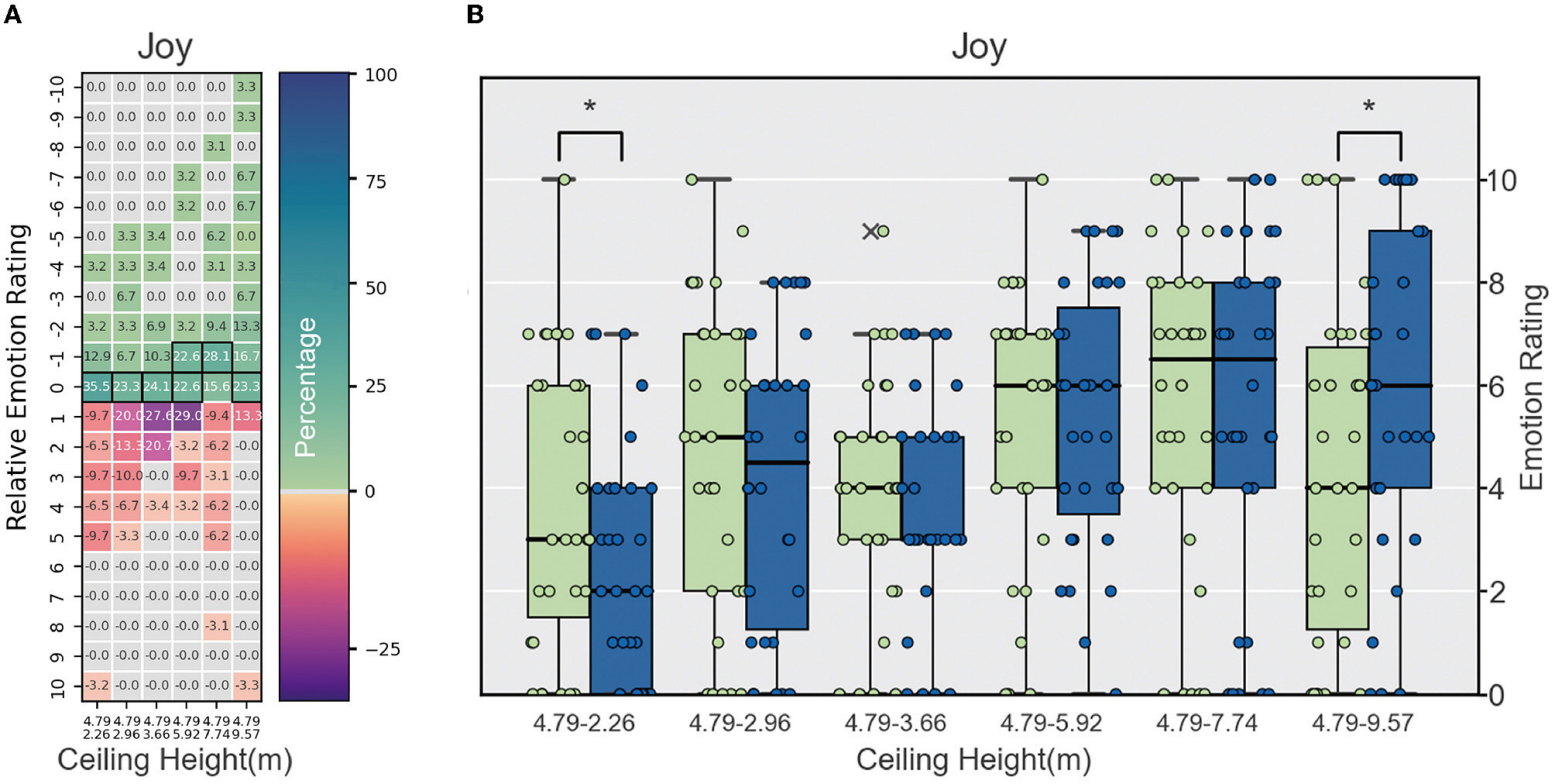
**(A)** Heatmap of relative emotion analysis for joy across ceiling heights, **(B)** box plot comparing joy responses at different ceiling heights to reference height, *p < 0.05.

1. Negative emotions:
• Fear: The fear scores varied among the groups, but no consistent pattern was found in relation to ceiling height. No significant differences existed between the reference height (4.79 m) and comparison heights (2.26, 2.96, 3.66, 5.92, 7.74, and 9.57m) in any groups, indicating that ceiling height may not have a substantial impact on fear levels. However, it is worth noting that the differences in the mean and median scores varied across groups, suggesting that other factors may be influencing the fear response in these environments.
• Anger: Similar to the fear category, the anger scores did not show a consistent pattern across ceiling heights. No significant differences were found between the reference height (4.79 m) and comparison heights (2.26, 2.96, 3.66, 5.92, 7.74, and 9.57 m) in any groups, indicating that ceiling height might not play a major role in the experience of anger. However, the variations in mean and median scores across the groups suggest that other contextual factors may have an impact on anger levels.
• Sadness: The results for sadness showed no clear relationship with ceiling height, with varying mean and median scores across the groups. No significant differences were found between the reference height (4.79 m) and comparison heights (2.26, 2.96, 3.66, 5.92, 7.74, and 9.57 m) in any group. This lack of consistency in the results suggests that other factors, besides ceiling height, might be influencing the experience of sadness in these environments.
• Disgust: The experience of disgust varied among groups, with some groups showing significant differences between the reference height (4.79 m) and comparison heights (2.26 m, 2.96 m). In the 2.26m and 2.96 m ceiling height groups, the Wilcoxon signed-rank test revealed significant differences (*p* = 0.00624 and *p* = 0.00757, respectively), with moderate effect sizes (0.2747 and 0.23888). The results indicate that ceiling height may have some influence on the disgust response, but the relationship is not consistent across all groups (see Figure 7).
2. Positive emotions:
• Joy: The relationship between ceiling height and joy demonstrated an interesting pattern. In rooms with ceiling heights of 2.26 and 9.57 m, significant differences were found between the reference height (4.79 m) and comparison heights (2.26 and 9.57 m), indicating that both very low and very high ceiling heights had an impact on the experience of joy. The 2.26 m ceiling height group showed a negative relationship, while the 9.57 m ceiling height group displayed a positive relationship. This suggests that the influence of ceiling height on joy is not linear, and there might be an optimal range where the difference is less pronounced. For the other ceiling heights (2.96, 3.66, 5.92, and 7.74 m), no significant differences were observed between the reference height (4.79 m) and comparison heights. This indicates that within this range of ceiling heights, the experience of joy is relatively stable and not significantly affected by ceiling height variations. It is worth noting that the impact of ceiling height on joy is more pronounced at the two extremes (2.26 and 9.57 m), suggesting that both very low and very high ceiling heights may have unique effects on the experience of joy in these environments (see Figure 8).
• Surprise: The surprise scores displayed a mixed pattern across the groups. While some groups showed moderate differences between the reference height (4.79 m) and comparison heights (2.26, 2.96, 3.66, 5.92, 7.74, and 9.57 m), no consistent relationship was found with ceiling height. In most groups, no significant differences were detected between the reference height (4.79 m) and comparison heights (2.26, 2.96, 3.66, 5.92, 7.74, and 9.57 m), suggesting that other factors may be contributing to the experience of surprise in these environments. This highlights the complexity of the relationship between ceiling height and emotional responses, as other contextual factors may be at play in shaping the experience of surprise.

### 4.3 Study 2: choose ceiling height from emotions

**Figure 9:**
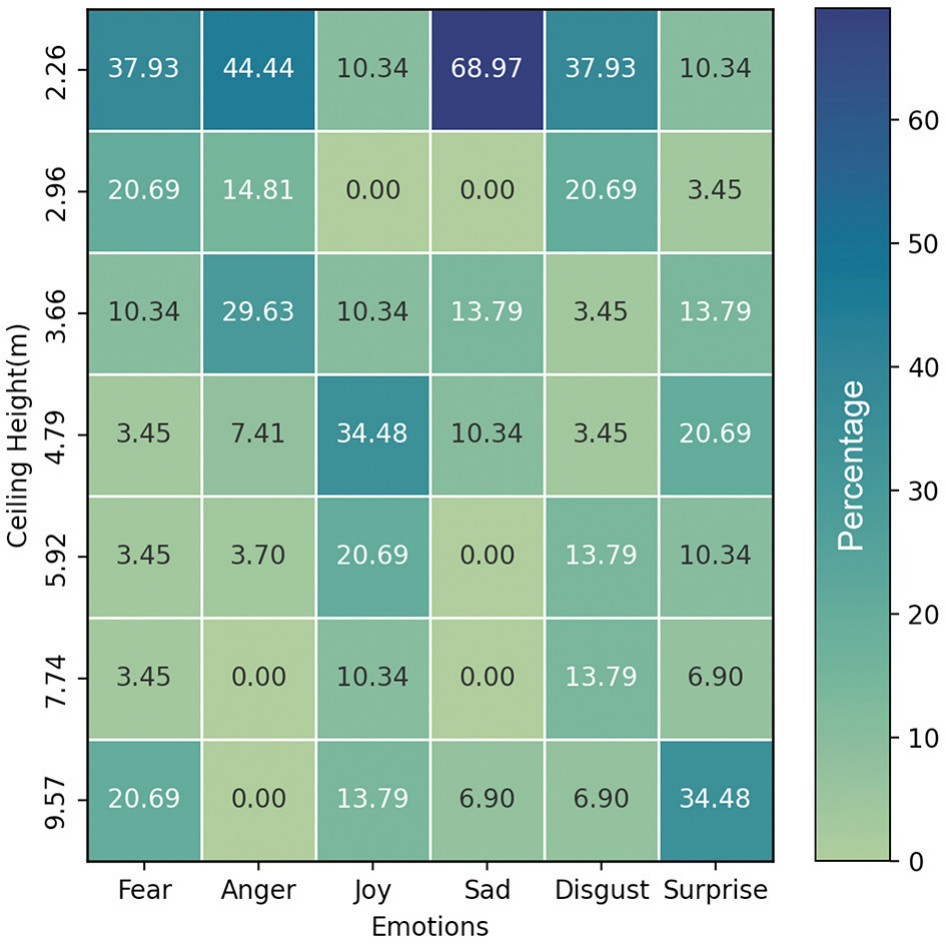
Heatmap of emotional associations with different ceiling heights in Study 2.

In Study 2, the results indicate that participants associated lower ceiling heights (2.26 and 2.96 m) with negative emotions such as fear, anger, sadness, and disgust. For instance, 37.93% of participants chose a 2.26 m ceiling height for fear, while 44.44% selected the same height for anger. On the other hand, a ceiling height of 4.79 m was predominantly chosen for the experience of joy, with 34.48% of participants selecting it. Interestingly, the highest ceiling height (9.57 m) elicited mixed responses, with 20.69% of participants choosing it for fear and 13.79% for joy (see Figure 9). This suggests that extreme ceiling heights may have unique effects on emotional experiences. The results for surprise were also varied, with no clear pattern emerging in relation to ceiling height.

## 5 Discussion

The findings from this study uncover a complex relationship between ceiling height and emotional responses, as demonstrated in both Study 1 and Study 2. While previous research broadly categorizes lower ceiling heights as fostering negative emotions and higher ceiling heights as promoting positive emotions (Vartanian et al., 2015; Cha et al., 2019), our study delves into more detailed emotional responses. In addition to the structured questionnaire responses, participants provided spontaneous verbal feedback during the main experiment, which indicated a clear perception of changes in ceiling heights. This feedback further validates our findings, demonstrating the effectiveness of our VR simulations in realistically portraying different architectural dimensions.

Specifically, our data indicate that lower ceiling heights correspond to increased feelings of disgust, while higher ceiling heights are linked to enhanced joy. However, the effects on other emotions such as fear, anger, sadness, and surprise are less evident. No consistent patterns emerged for these emotions when comparing participants' responses to the reference height of 4.79 meters.

Upon further analysis, significant differences were observed for disgust between the reference height and the lower comparison heights of 2.26 and 2.96 m. The relationship between ceiling height and joy also presented a distinct pattern, with notable differences identified at the lowest (2.26 m) and highest (9.57 m) ceiling heights. These findings underscore the need for a more detailed understanding of emotional responses to ceiling heights in architectural spaces.

In Study 2, the results indicated that participants associated lower ceiling heights with negative emotions, such as fear, anger, sadness, and disgust, while a ceiling height of 4.79 m was predominantly chosen for experiencing joy. The highest ceiling height (9.57 m) elicited mixed responses, with participants selecting it for both fear and joy, suggesting that extreme ceiling heights may have unique effects on emotional experiences. The results for surprise were varied, with no discernible pattern emerging concerning ceiling height.

Our research differs from the previous studies in several ways, although our findings partially align with theirs. We implemented a wider variety of ceiling heights and provided more precise emotional categorizations. Initially, we considered using a questionnaire format similar to the Self-Assessment Manikin (SAM) (Bradley and Lang, 1994), but such questionnaires can be challenging to translate into clear and straightforward terms, making the results less accessible to non-psychology professionals.

Our second hypothesis, which proposed the use of editable 360-degree panoramic scenes for simulating architectural settings, appears to have successfully provided a more ecologically valid and comprehensive understanding of the relationship between ceiling height and emotional responses. However, to better capture the nuances of real-world architectural experiences, advancements in technology are necessary, such as the integration of Building Information Modeling (BIM) and Augmented Reality (AR) techniques (Chai et al., 2020). The use of BIM-AR technology could potentially enhance the realism of virtual environments and offer a more accurate representation of how individuals interact with and respond to architectural spaces in real-world settings.

Our research offers significant implications for the design of various architectural spaces and contributes to the field of environmental psychology. The nuanced understanding of how ceiling heights affect emotional responses can inform architects and designers in their decision-making process. This is particularly relevant for spaces where emotional ambiance is crucial, such as in healthcare settings, educational environments, and commercial spaces. For instance, higher ceilings in creative spaces could foster an atmosphere of openness and innovation, while lower ceilings in residential settings might create a sense of coziness and security.

Additionally, these findings enrich our understanding within environmental psychology of how physical space influences human emotions. They provide a tangible link between architectural design elements and emotional wellbeing, offering a new perspective on creating spaces that not only meet physical needs but also support emotional health.

Furthermore, the potential of editable 360-degree panoramic scenes in architectural design and virtual reality demonstrates a promising tool for pre-testing the emotional impact of space. This technology could become integral in future architectural planning and design, allowing for a more user-centered approach that prioritizes emotional responses.

## 6 Limitations

Our study does present several limitations. Firstly, the participant pool consisted solely of architecture students, which may have influenced their perception of the virtual environments and subsequent emotional responses. Moreover, conducting the study within a virtual art gallery setting might not fully replicate the nuanced experiences of real-world architecture. However, it's worth noting that the virtual environment does offer certain advantages, including the ability to maintain consistency and control over experimental conditions.

Secondly, while our study has provided insights into the relationship between ceiling height and emotional responses, it's important to recognize that this relationship might also be influenced by other spatial factors, such as room size and width. Our investigation was confined to a single space, and future research could further explore this dynamic under varied spatial conditions. Regarding the sequence of experimental conditions, we gave this careful consideration during the design phase of the study. The decision to use a medium ceiling height as the reference condition was informed by its common prevalence in many architectural settings, providing a relatable starting point for our participants. During the experiment, several participants even questioned whether they were entering a room of the same height when transitioning to the mid-height conditions, thereby inadvertently validating our decision. We recognize that altering the sequence of experiences could potentially affect the results. For instance, if participants were exposed to the extremes of ceiling height first, it might prime their subsequent responses. As such, future studies might consider manipulating the order of conditions to explore its impact on emotional responses. Nonetheless, for the purposes of the current study, we maintain that our approach allows for a more consistent and controlled exploration of the impact of varying ceiling heights on emotions.

In terms of evaluating emotional responses, our study utilized self-reported measures (Robins et al., 2007). Future studies could consider implementing more objective measurement methods, such as facial expression recognition, which we are currently developing, to provide a more comprehensive assessment of participants' emotional experiences.

These limitations notwithstanding, our findings offer valuable insights into the effects of architectural dimensions on human emotions. This understanding can serve as a foundation for further research in architectural design and environmental psychology, contributing to the creation of more empathetic and emotionally resonant spaces.

## 7 Conclusion

In this study, we explored the intricate relationship between ceiling height and emotional responses within art gallery settings. Our key findings demonstrate that varying ceiling heights significantly influence emotional experiences, particularly affecting feelings of joy and disgust. The use of editable 360-degree panoramic photo scene modeling technology proved effective in simulating these variations, offering valuable insights into the impact of spatial dimensions on emotional responses.

This research contributes to the broader understanding of how architectural features can shape human emotions, highlighting the importance of considering spatial dimensions in architectural design. The methodology employed in this study opens up new avenues for investigating various architectural aspects and their emotional implications, offering promising prospects for future research in architectural design and environmental psychology.

Our findings provide a foundation for future studies to further explore the complex relationship between architectural spaces and human emotional experiences, ultimately guiding the creation of spaces that enhance wellbeing and emotional comfort.

## Data availability statement

The datasets presented in this study can be found in online repositories. The names of the repository/repositories and accession number(s) can be found at: 10.6084/m9.figshare.22634017.

## Ethics statement

The studies involving humans were approved by Polytechnic University of Catalonia Ethics Committee. The studies were conducted in accordance with the local legislation and institutional requirements. The participants provided their written informed consent to participate in this study.

## Author contributions

ZZ: Conceptualization, Formal analysis, Methodology, Software, Visualization, Writing – original draft. JF: Conceptualization, Methodology, Supervision, Writing – review & editing. LM: Conceptualization, Data curation, Methodology, Supervision, Writing – review & editing. YC: Investigation, Writing – review & editing.
